# Association between chronic obstructive pulmonary disease and gastroesophageal reflux disease: a national cross-sectional cohort study

**DOI:** 10.1186/1471-2466-13-51

**Published:** 2013-08-09

**Authors:** Jinhee Kim, Jin Hwa Lee, Yuri Kim, Kyungjoo Kim, Yeon-Mok Oh, Kwang Ha Yoo, Chin Kook Rhee, Hyoung Kyu Yoon, Young Sam Kim, Yong Bum Park, Sei Won Lee, Sang Do Lee

**Affiliations:** 1 Office of Heath Service Research, National Evidence-Based Healthcare Collaborating Agency, Seoul, Korea; 2 Department of Internal Medicine, School of Medicine, Ewha Womans University, Seoul, Korea; 3 Department of Clinical Research Support, National Strategic Coordinating Center for Clinical Research, Seoul, Korea; 4 Department of Pulmonary and Critical Care Medicine, Asan Medical Center, University of Ulsan College of Medicine, Seoul, Korea; 5 Department of Internal Medicine, Konkuk University School of Medicine, Seoul, Korea; 6 Department of Internal Medicine, Seoul St. Mary’s Hospital, Catholic University of Korea, Seoul, Korea; 7 Department of Internal Medicine, Yeouido St. Mary’s Hospital, Catholic University of Korea, Seoul, Korea; 8 Department of Internal Medicine, Yonsei University College of Medicine, Seoul, Korea; 9 Department of Internal Medicine, Division of Pulmonary, Allergy, and Critical Care Medicine, Hallym University Kangdong Sacred Heart Hospital, Seoul, Korea

**Keywords:** Gastroesophageal reflux, Obstructive lung disease, Prevalence, Exacerbation, Anticholinergic

## Abstract

**Background:**

Gastroesophageal reflux disease (GERD) is one of the most common causes of chronic cough and a potential risk factor for exacerbation of chronic obstructive pulmonary disease (COPD). The aim of this study was to investigate the prevalence and risk factors of GERD in patients with COPD and association between GERD and COPD exacerbation.

**Methods:**

Data were collected from the National Health Insurance Database of Korea. The subjects were 40 years old and older, who had COPD as primary or secondary diagnosis codes and utilized health care resource to receive prescriptions of COPD medication at least twice in 2009. Univariate logistic regression was performed to understand the relationship between COPD and GERD, and multiple logistic regression analysis was performed with adjustment for several confounding factors.

**Results:**

The prevalence of GERD in COPD patients was 28% (39,987/141,057). Old age, female gender, medical aid insurance type, hospitalization, and emergency room (ER) visit were associated with GERD. Most of COPD medications except inhaled muscarinic antagonists were associated with GERD. The logistic regression analysis showed that the presence of GERD was associated with increased risk of hospitalization (OR 1.54, CI 1.50 to 1.58, p<0.001) and frequent ER visits (OR 1.55, CI 1.48 to 1.62, p<0.001).

**Conclusions:**

The prevalence of GERD in patients with COPD was high. Old age, female gender, medical aid insurance type, and many COPD medications except inhaled muscarinic antagonists were associated with GERD. The presence of GERD was associated with COPD exacerbation.

## Background

Exacerbations of chronic obstructive pulmonary disease (COPD) have a significant impact on patient quality of life and can accelerate lung function decline [1], which is associated with increased morbidity and mortality [2,3]. Gastroesophageal reflux disease (GERD) is one of the most common causes of chronic cough [4] and a potential risk factor for exacerbation of COPD [5-7]. GERD is a relatively common condition, affecting 10–29% of the western population [8] and 3–12% of Korean adults [9]. GER can heighten bronchial reactivity and microaspiration [10-13]. Abnormal GER was objectively assessed and clearly associated to lung diseases [14,15]. Laryngopharyngeal sensitivity is important in preventing pulmonary aspiration. Patients with cough and GERD have significantly reduced laryngopharyngeal sensitivity to air stimuli compared with healthy subjects [16]. COPD patients have flat diaphragm and increased intra-abdominal and negative intra-thoracic pressure, which could aggravate GER [17]. In addition, medications such as theophylline and inhaled beta-2 agonists may decrease the lower esophageal sphincter pressure, could facilitate GER [18,19]. Therefore several small studies showed that GERD is more common in patients with COPD than that in those without COPD [20-22]. Also it has been suggested that an increase in the frequency of COPD exacerbation can be associated with the presence of GERD [6,7,23,24].

We used the National Health Insurance Database of Korea and tried to answer three questions. First question was the prevalence of GERD in patients with COPD. Second one was which factors are associated with GERD in COPD patients. Third was whether the presence of GERD is associated with exacerbation of COPD even after adjusting confounding factors.

## Methods

### Data source and ethical considerations

The present study used the National Health Insurance claims data for medical services provided to Koreans aged ≥ 40 years between January 1, 2009 and December 31, 2009. These data were provided by the Korean Health Insurance Review and Assessment Service (HIRA), which was established to review claims data and assess healthcare in Korea and is under the responsibility of a single agency independent from insurers, providers, and other interested parties. All Koreans are covered under the national health insurance system, and the HIRA database contains all information regarding submitted claims and prescriptions.

The HIRA claims database comprises general item, medical treatment, diagnosis, and prescription databases. The general item database contains information such as patient identifier, age, sex, and insurance type; hospital name, type, location, and medical department; and medical cost, number of days hospitalized and/or receiving outpatient, intensive care unit (ICU), and/or emergency room (ER) care. The medical treatment database contains information such as patient identifier, detailed medical treatment records, and drug administration (brand name, total days of medication, single dose, daily dose, and unit cost). The diagnosis database contains information such as patient identifier, main diagnosis, and submain diagnoses, recorded according to the International Classification of Diseases, 10th Revision (ICD-10). The prescription database contains information such as patient identifier and outpatient prescriptions. All databases were linked using a mutual identifier.

The National Evidence-Based Healthcare Collaborating Agency (NECA) ethics committee approved the present study (PIRB11-022).

### Study population

The subjects of the current study were patients aged ≥40 years with COPD recorded as ICD-10 codes J42.x–J44.x (except J430) for the principal or additional diagnoses who were given prescriptions including at least one of the following medications (including oral, injected, or inhaled), at least twice per year: combinations in a single inhaler of inhaled corticosteroids (ICSs) and long-acting beta-2 agonists (LABAs), long-acting muscarinic antagonists (LAMAs), LABAs, short-acting muscarinic antagonists (SAMAs), inhalers with both short-acting beta-2 agonists (SABAs) and SAMAs, oral beta-2 agonists, or theophylline.

Patients with more than one claim per year due to cancer (C00.x–C97.x) and/or cerebrovascular disease (I60.x–I69.x) were excluded from this study because the enormous expenses claimed for treatment of these diseases were difficult to distinguish from COPD- and GERD-related medical services. Additionally, through consultation with six physicians of pulmonary medicine, we decided to exclude patients with more than one claim per year for hiatal hernia (K449), gastric surgery (K910–K913), Zollinger-Ellison syndrome (E164), systemic sclerosis (M34), achalasia (K220), pyloric obstruction (K311–K315), obesity (E66.x), alcoholic liver disease (K70.x), or ascites (K7151, R18, C786, A1830) because these diseases increase the risk for GERD.

Among subjects in the present study, 39,987 patients with COPD used medical services more than once per year for GERD (K21, K210, K219), and 101,070 patients with COPD did not have GERD.

### Measures

#### Baseline characteristics

The variables considered to be baseline characteristics were sex, age, insurance type, hospitalization and ICU hospitalization, number of ER visits, presence of chest X-rays, spirometry, chest CT, esophagogastroduodenoscopy, 24-h esophageal pH monitoring and esophagography, COPD severity, and comorbidity. The ‘severe’ COPD group comprised patients who visited a tertiary medical institution and were prescribed ICS + LABA + LAMA, ICS + LABA + oral corticosteroid (OCS), or LAMA + OCS more than once per year, and the ‘not severe’ group comprised the remaining study subjects. ER visits were defined as cases in which an outpatient or hospitalization claim was made for emergency medical service fees or in which additional holiday/night service fees were incurred. Co-morbidities included ischemic heart disease (I20.x–I25.x, except I20.1), osteoporosis (M80.x–M82.x), depressive disorder (F32.x–F33.x), arthritis (M05.x–M09.x, M13.x), diabetes mellitus (E10.x–E14.x), congestive heart failure (I50, I50.0, I50.1, I50.9), hypertension (I10.x), anemia (D50.x–D53.x, D63.x), metabolic syndrome (E66, E78.0, E78.4, E78.5), Barrett’s esophagus (B227), gastric ulcer (K25.x), duodenal ulcer (K26.x), peptic ulcer (K27.x–K28.x), and gastroduodenitis (K29.x).

#### Medications used

Medications considered in the present study included ICSs, ICSs/LABAs, LAMAs, LABAs, leukotriene receptor antagonists (LTRAs), OCSs, SAMAs, SABAs, SABAs/SAMAs, oral beta-2 agonists, and theophylline covering all form of drug such as oral medications, injections, and inhalations.

#### Exacerbation of COPD

Exacerbation was defined as hospitalization or ER visits. Only subjects who were admitted to the department of internal medicine and received systemic corticosteroids during at least three days or recorded as ICD code for COPD exacerbation were included. Cases receiving systemic corticosteroids and inhaled short-acting bronchodilators at ER visits were included.

### Statistical analyses

Data were analyzed using the SAS statistical program (ver. 9.2 [SAS Institute]); details of the analytical methods were as follows.

First, frequencies and percentages were calculated to enable comparison of baseline characteristics between COPD patients with GERD and those without GERD; the chi-squared test was used for categorical data, and the *t*-test was used for continuous data.

Second, the difference in medication usage between groups was determined using frequencies and percentages.

Third, univariate logistic regression was performed to understand the relationship between the general characteristics of patients with COPD and the presence of GERD, and multiple logistic regression was performed, with general characteristic variables included in the model.

Fourth, univariate logistic regression was performed to understand the relationship between the co-morbidity of patients with COPD and the presence of GERD, and the relationship between the medication usage of patients with COPD and the presence of GERD. Additionally, multiple logistic regression analysis was performed with adjustment for sex, age, type of health insurance, history of hospitalization and ICU hospitalization, category of ER visit, and COPD severity.

Fifth, univariate logistic regression was performed to understand the relationship between the presence of GERD in patients with COPD and the exacerbation of COPD, and multiple logistic regression was performed with adjustment for sex, age, type of health insurance, and COPD severity.

## Results

The prevalence of GERD in patients with COPD was 28% (39,987/141,057).

General characteristics of patients with COPD according to the presence or absence of GERD were summarized in Table 1. COPD patients with GERD had more hospitalization and ER visits than those without GERD (all *p* < 0.001), whereas there was no difference of ICU hospitalization between two groups. More patients with COPD and GERD used medical services for treatment of all kinds of comorbidity than did those without GERD (all *p* < 0.001; Table 1).

**Table 1:** General characteristic of subjects with COPD, classified according to the presence of GERD

	**Variables**	**COPD with GERD**		**COPD without GERD**		***p***
		**No.**	**(%)**	**No.**	**(%)**	
**Total**		39,987	(100.0)	101,070	(100.0)	
**Sex**						<0.001
	Male	22,967	(57.4)	61,674	(61.0)	
	Female	17,020	(42.6)	39,396	(39.0)	
**Age (years)**						
	Mean ± SD	68.86 ± 10.18		69.71 ± 11.67		<0.001
	40–49	1,904	(4.8)	5,243	(5.2)	<0.001
	50–59	5,349	(13.4)	13,052	(12.9)	
	60–69	11,954	(29.9)	26,801	(26.5)	
	70–79	15,239	(38.1)	37,329	(36.9)	
	80+	5,541	(13.9)	18,645	(18.4)	
**Type of insurance**						<0.001
	Health insurance	32,823	(82.1)	85,755	(84.8)	
	Medical aid	7,164	(17.9)	15,315	(15.2)	
**Hospitalization**						<0.001
	Never	24,179	(60.5)	70,547	(69.8)	
	Ever	15,808	(39.5)	30,523	(30.2)	
**ICU hospitalization**						0.377
	Never	39,817	(99.6)	100,605	(99.5)	
	Ever	170	(0.4)	465	(0.5)	
**No. of ER visits**						
	Mean ± SD	1.65 ± 1.24		1.50 ± 1.02		<0.001
	0	31,260	(78.2)	84,116	(83.2)	<0.001
	1–2	7,446	(18.6)	15,121	(15.0)	
	3–4	984	(2.5)	1,506	(1.5)	
	5+	297	(0.7)	327	(0.3)	
**Chest X-ray**						<0.001
	Never	22,671	(56.7)	65,494	(64.8)	
	Ever	17,316	(43.3)	35,576	(35.2)	
**Spirometry**						<0.001
	Never	23,353	(58.4)	65,891	(65.2)	
	Ever	16,634	(41.6)	35,179	(34.8)	
**Chest CT**						0.055
	Never	39,583	(99.0)	100,159	(99.1)	
	Ever	404	(1.0)	911	(0.9)	
**Esophagogastroduodenoscopy**						<0.001
	Never	27,923	(69.8)	94,638	(93.6)	
	Ever	12,064	(30.2)	6,432	(6.4)	
**24-h esophageal pH monitoring**						<0.001
	Never	39,974	(100.0)	101,067	(100.0)	
	Ever	13	(0.0)	3	(0.0)	
**Esophagography**						<0.001
	Never	39,352	(98.4)	100,566	(99.5)	
	Ever	635	(1.6)	504	(0.5)	
**COPD severity**						<0.001
	No	32,629	(81.6)	84,427	(83.5)	
	Yes	7,358	(18.4)	16,643	(16.5)	
**Comorbidity**						
	Ischemic heart disease	8,262	(20.7)	15,696	(15.5)	<0.001
	Osteoporosis	8,723	(21.8)	15,281	(15.1)	<0.001
	Depressive disorder	4,838	(12.1)	6,442	(6.4)	<0.001
	Arthritis	10,299	(25.8)	18,035	(17.8)	<0.001
	Diabetes mellitus	10,426	(26.1)	22,478	(22.2)	<0.001
	Congestive heart failure	3,819	(9.6)	8,661	(8.6)	<0.001
	Hypertension	20,734	(51.9)	48,802	(48.3)	<0.001
	Anemia	2,354	(5.9)	3,973	(3.9)	<0.001
	Metabolic syndrome	9,296	(23.2)	16,428	(16.3)	<0.001
	Barrett’s esophagus	23	(0.1)	4	(0.0)	<0.001
	Gastric ulcer	14,308	(35.8)	17,940	(17.8)	<0.001
	Duodenal ulcer	2,489	(6.2)	2,404	(2.4)	<0.001
	Peptic ulcer	8,856	(22.1)	13,657	(13.5)	<0.001
	Gastroduodenitis	36,196	(90.5)	77,151	(76.3)	<0.001

No. = number of subjects; SD = standard deviation; *ICU* = intensive care unit; *ER* = emergency room.

Medication used for both group was summarized in Table 2.

**Table 2:** Medication utilization of patients with COPD, classified according to the presence of GERD

	**COPD with GERD (*n*= 39,987)**		**COPD without GERD (*n*= 101,070)**	
	**No.**	**(%)**	**No.**	**(%)**
**ICS**	6,439	(8.8)	12,734	(7.7)
**ICS/LABA**	14,436	(19.6)	33,593	(20.3)
**LAMA**	11,760	(16.0)	28,529	(17.2)
**LABA**	35	(0.0)	79	(0.0)
**LTRA**	11,468	(15.6)	23,158	(14.0)
**OCS**	24,031	(32.7)	51,421	(31.0)
**SAMA**	7,763	(10.6)	16,850	(10.2)
**SABA**	14,596	(19.9)	32,904	(19.9)
**SABA/SAMA**	1,048	(1.4)	2,603	(1.6)
**Oral beta-2 agonists**	23,530	(32.0)	55,856	(33.7)
**Theophylline**	33,859	(46.0)	83,688	(50.5)

No. = number of subjects; *ICS* = inhaled corticosteroid; *LABA* = long-acting beta-2 agonist; *LAMA* = long-acting muscarinic antagonist; *LTRA* = leukotriene receptor antagonist; *OCS* = oral corticosteroid; *SAMA* = short-acting muscarinic antagonist; *SABA* = short-acting beta-2 agonist.

A regression model including general characteristics indicated that more female than male patients with COPD had GERD and more patients in their 50s, 60s, and 70s had GERD compared with those in their 40s. More GERD was observed in the medical aid group compared with the health insurance group, and in subjects with hospitalization experience compared with subjects without hospitalization. Less GERD was observed in subjects with ICU hospitalization than in those without. More GERD were observed in subjects with ER visits compared with those without (Table 3).

**Table 3:** Association of GERD with general characteristics in patients with COPD

		**Unadjusted**			Adjusted*		
		**OR**	**95% CI**	***p***	**OR**	**95% CI**	***p***
**Sex**							
	Male	1 (ref)			1 (ref)		
	Female	1.16	(1.13, 1.19)	<0.001	1.22	(1.19, 1.25)	<0.001
**Age (years)**							
	40–49	1 (ref)			1 (ref)		
	50–59	1.13	(1.06, 1.20)	<0.001	1.15	(1.08, 1.22)	<0.001
	60–69	1.23	(1.16, 1.30)	<0.001	1.26	(1.19, 1.34)	<0.001
	70–79	1.12	(1.06, 1.19)	<0.001	1.10	(1.04, 1.17)	<0.001
	80+	0.82	(0.77, 0.87)	<0.001	0.75	(0.71, 0.80)	<0.001
**Type of insurance**							
	Health insurance	1 (ref)			1 (ref)		
	Medical aid	1.22	(1.19, 1.26)	<0.001	1.15	(1.12, 1.19)	<0.001
**Hospitalization**							
	Never	1 (ref)			1 (ref)		
	Ever	1.51	(1.48, 1.55)	<0.001	1.46	(1.42, 1.50)	<0.001
**ICU hospitalization**							
	Never	1 (ref)			1 (ref)		
	Ever	0.93	(0.78, 1.10)	0.385	0.68	(0.57, 0.82)	<0.001
**Number of ER visits**							
	0	1 (ref)			1 (ref)		
	1–2	1.33	(1.29, 1.37)	<0.001	1.11	(1.07, 1.15)	<0.001
	3–4	1.76	(1.62, 1.91)	<0.001	1.40	(1.29, 1.53)	<0.001
	5+	2.44	(2.09, 2.86)	<0.001	1.87	(1.59, 2.21)	<0.001
**COPD severity**							
	No	1 (ref)			1 (ref)		
	Yes^†^	1.14	(1.11, 1.18)	<0.001	1.02	(0.99, 1.06)	0.186

*OR* = odds ratio; *ICU* = intensive care unit; *ER* = emergency room.

^*^Adjusted for sex, age, type of insurance, hospitalization, ICU hospitalization, number of ER visits by category, and COPD severity.

^†^The ‘severe’ group comprised patients who visited a tertiary medical institution and were prescribed ICS + LABA + LAMA, ICS + LABA + oral corticosteroid (OCS), or LAMA + OCS more than once per year.

After adjusting for sex, age, type of health insurance, hospitalization, ICU hospitalization, category of ER visit, and COPD severity, more patients with COPD and GERD had comorbidities except congestive heart failure. More GERD was observed among patients using ICSs, ICSs/LABAs, LTRAs, OCSs, oral beta-2 agonists, and theophylline (all *p* < 0.001). However, less GERD was observed in association with SAMAs use [odds ratio (OR) 0.96, 95% confidence interval (CI) 0.93 to 0.99; Table 4].

**Table 4:** Association of GERD with comorbidities and medication utilization in patients with COPD

		**Unadjusted**			Adjusted*		
		**OR**	**95% CI**	***p***	**OR**	**95% CI**	***p***
**Comorbidity**							
	Ischemic heart disease	1.42	(1.38, 1.46)	<0.001	1.31	(1.27, 1.35)	<0.001
	Osteoporosis	1.57	(1.52, 1.61)	<0.001	1.46	(1.42, 1.51)	<0.001
	Depressive disorder	2.02	(1.95, 2.10)	<0.001	1.87	(1.79, 1.94)	<0.001
	Arthritis	1.60	(1.55, 1.64)	<0.001	1.55	(1.51, 1.60)	<0.001
	Diabetes mellitus	1.23	(1.20, 1.27)	<0.001	1.14	(1.11, 1.17)	<0.001
	Congestive heart failure	1.13	(1.08, 1.17)	<0.001	1.04	(0.99, 1.08)	0.078
	Hypertension	1.15	(1.13, 1.18)	<0.001	1.11	(1.09, 1.14)	<0.001
	Anemia	1.53	(1.45, 1.61)	<0.001	1.39	(1.32, 1.46)	<0.001
	Metabolic syndrome	1.56	(1.52, 1.61)	<0.001	1.45	(1.41, 1.49)	<0.001
	Barrett’s esophagus	14.46	(5.01, 41.73)	<0.001	15.10	(5.21, 43.79)	<0.001
	Gastric ulcer	2.58	(2.52, 2.65)	<0.001	2.45	(2.38, 2.51)	<0.001
	Duodenal ulcer	2.72	(2.57, 2.88)	<0.001	2.65	(2.51, 2.81)	<0.001
	Peptic ulcer	1.82	(1.77, 1.88)	<0.001	1.74	(1.69, 1.79)	<0.001
	Gastroduodenitis	2.96	(2.85, 3.07)	<0.001	2.82	(2.71, 2.92)	<0.001
**Drug**							
	ICS	1.33	(1.29, 1.38)	<0.001	1.13	(1.09, 1.17)	<0.001
	ICS/LABA	1.14	(1.11, 1.16)	<0.001	1.05	(1.02, 1.08)	<0.001
	LAMA	1.06	(1.03, 1.09)	<0.001	0.99	(0.96, 1.02)	0.567
	LABA	1.12	(0.75, 1.67)	0.568	1.11	(0.74, 1.65)	0.624
	LTRA	1.35	(1.31, 1.39)	<0.001	1.27	(1.23, 1.30)	<0.001
	OCS	1.45	(1.42, 1.49)	<0.001	1.37	(1.34, 1.40)	<0.001
	SAMA	1.20	(1.17, 1.24)	<0.001	0.96	(0.93, 0.99)	0.015
	SABA	1.19	(1.16, 1.22)	<0.001	1.02	(0.99, 1.04)	0.237
	SABA/SAMA	1.02	(0.95, 1.10)	0.627	0.97	(0.90, 1.04)	0.339
	Oral beta-2 agonists	1.16	(1.13, 1.19)	<0.001	1.11	(1.09, 1.14)	<0.001
	Theophylline	1.15	(1.11, 1.19)	<0.001	1.10	(1.07, 1.14)	<0.001

*OR* = odds ratio; *ICS* = inhaled corticosteroid; *LABA* = long-acting beta-2 agonist; *LAMA* = long-acting muscarinic antagonist; *LTRA* = leukotriene receptor antagonist; *OCS* = oral corticosteroid; *SAMA* = short-acting muscarinic antagonist; *SABA* = short-acting beta-2 agonist.

^*^Adjusted for sex, age, type of health insurance, hospitalization, ICU hospitalization, number of ER visits by category, and COPD severity.

After adjusting for sex, age, type of health insurance, and COPD severity, the regression model demonstrated that COPD exacerbation was more prevalent among patients with GERD than among those without GERD, as indicated by more hospitalization (OR 1.54, 95% CI 1.50 to 1.58) and ER visits (OR 1.55, 95% CI 1.48 to 1.62; Table 5).

**Table 5:** Association of GERD with exacerbation in patients with COPD

		**Hospitalization**			**ICU**			**ER visit (0–1*vs*. ≥2)**		
		**OR**	**95% CI**	***p***	**OR**	**95% CI**	***p***	**OR**	**95% CI**	***p***
**GERD**	No	1 (ref)			1 (ref)			1 (ref)		
	Yes	1.51	(1.48, 1.55)	<0.001	0.92	(0.78, 1.10)	0.377	1.55	(1.48, 1.62)	<0.001
GERD*	No	1 (ref)			1 (ref)			1 (ref)		
	Yes	1.54	(1.50, 1.58)	<0.001	0.93	(0.78, 1.11)	0.441	1.55	(1.48, 1.62)	<0.001

*ICU* = intensive care unit; *ER* = emergency room; *OR* = odds ratio.

^*^Adjusted for sex, age, type of health insurance, and COPD severity.

## Discussion

To the best of our knowledge, this is the first nationwide study of the largest number of COPD patients to investigate the prevalence of GERD and the association between COPD and GERD. The prevalence of GERD in patients with COPD was 28%, which is very high since the prevalence in Korean general population is around 12%. It is similar to previous ones reported in Japan [7,24], and lower than the others, which were 32%–37% in the USA and 53.6% in Iran [6,20,23], even though these studies investigated only a small number of patients with COPD. Some of them showed higher prevalence in COPD patients compared with controls [7,20]. These support that GERD is one of the most common comorbidities in patients with COPD.

In the present study, several things are different from the previous studies. One is that inhaled anticholingergics may decrease or, at least, not increase risk of GERD, whereas a previous study reported that relative risk of GERD increased with using inhaled anticholinergics [22]. In another study, the effect of medication was not statistically significant or hard to be evaluated because COPD patients are usually treated by multiple medications and have both pulmonary and other systemic problems [21]. Our hypothesis is that inhaled anticholinergics might reduce GER through suppressing cough. They have been used for chronic cough [25], which is one of the most common symptoms in COPD patients. Chronic bronchitis and GERD are the most common causes of chronic cough [26]. Ipratropium has been effective even for cough hypersensitivity induced by upper respiratory tract infection [27]. Tiotropium inhibits cough reflex sensitivity to capsaicin in subjects with acute viral upper respiratory tract infection [28]. The antitussive effect of tiotropium may occur through a mechanism other than bronchodilation. Additionally, tiotropium reduced lung inflammation in a mouse model of chronic GER [29]. Even though ICSs have been known to be effective for chronic cough, especially asthma [26], our subjects exclusively had COPD. The prevalence of GER was reported to increase according to use of ICS in COPD patients [22], which is consistent with our result. Combined inhalers of ICS and LABA have been popular in Korea, whereas use of LABA alone was very limited because representative LABAs such as salmeterol or formoterol were no longer imported or produced. The reason was that salmeterol as a single agent without ICS seemed to increase respiratory mortality in patients with asthma [30], which made physicians hesitate to prescribe LABA without ICS even for COPD. Therefore it is inadequate to evaluate their effects on GERD. When a patient takes variable drugs at the same time, as well as COPD medication, drug interaction will happen.

Another different thing is that ICU admission due to COPD exacerbation was not associated with GERD. It may be possible for COPD patients with GERD to complain more cough or heartburn and feel exacerbation earlier than do those without GERD, which make them more frequently have unscheduled visit or ER visit, leading to hospitalization to general ward but not to ICU. The other possibility is that patients with COPD to be admitted to ICU do not have chance to be examined whether they have GERD or they are too dyspneic to present any symptoms of GERD. Despite of higher proportion of comorbidities in patients with COPD and GERD, the rate of ICU admission was lower than those in those without GERD, even though total hospitalization rate was higher. There would be the third possibility. In this study, prescriptions of proton pump inhibitors (PPIs) were given for COPD patients with GERD more frequently than those without GERD (data are not shown). PPIs decrease frequency of GERD symptoms such as cough [31], which would rather prevent COPD patients from progression to ICU admission. One small prospective study demonstrated that PPIs reduced the number of exacerbation in COPD patients without GERD [32].

In this study, the logistic regression analysis, even after adjusting several factors such as age, gender, type of health insurance, and COPD severity, showed that the presence of GERD was independently associated with COPD exacerbation such as hospitalization or frequent ER visits. Since lung function data were not available in the present study, definition of severe COPD was based upon medication information and health care resource utilization. Although the degree of airflow limitation has been used as one of the most important criteria of COPD severity, it does not always reflect dyspnea, exercise capacity, quality of life, or exacerbation frequency [33]. Recently updated Global initiative for chronic Obstructive Lung Disease (GOLD) emphasizes combined assessment of COPD, which includes symptoms and risk of exacerbations as well as degree of airflow limitation [3]. To supplement our weakness, patients with severe COPD were defined as those who satisfied both of the followings. One is that they used a tertiary health care resource, which means respiratory specialist’s care, and the other is that they received medications for maximum effect such as triple therapy (ICS + LABA + LAMA) or OCS-containing long-acting bronchodilator, which may mean medication for exacerbations. These are possible under nationwide health insurance system of Korea, which divide health care resources into primary, secondary and tertiary and recommend people to use those step by step. Previous studies observed very small number of patients and simply compared frequency of exacerbation in COPD patients with GERD with in those without GERD [6,7,23,24]. They did not fully consider clinically important confounding factors such as age, gender, and COPD severity. Moreover, in two reports of them, COPD patients with GERD showed lower lung function as well as higher frequency of exacerbation than did those without GERD [23,24]. Therefore it might be hard to conclude whether frequent exacerbation was related to the presence of GERD or severe airflow limitation. Our study is also limited by its retrospective and cross-sectional design, and thus we cannot definitely conclude that GERD causes exacerbations of COPD. However, in a recent prospective study, patients with COPD and GERD had more exacerbation and hospitalization than in those without GERD, with crude relative risks of 3.42 and 3.66, respectively [24]. Since they excluded patients taking drugs for acid suppression, their relative risks may be higher than odds ratio in this study reflecting real world.

There are several limitations of this study. GERD was defined only by diagnosis codes even though about one third of GERD patients performed diagnostic tests related to GERD. In Korea, diseases of upper gastrointestinal (UGI) tract are more common than those in western countries and the incidence of stomach cancer has been the highest among all types of cancer. Therefore, as a health screening program of the Korean National Health Insurance System, all people can receive one of EGD or UGI series every two years from the age of 40 years. Nevertheless there might be a selection bias since symptomatic patients are likely to receive a diagnostic test. Definition of COPD was based on both of diagnosis codes and prescription data, which were exclusively determined by physicians. Although 37% of our subjects performed spirometry, this figure includes both general physicians and specialists. To exclude asthma patients, we excluded those receiving only LTRA or only ICS or only SABA. Nevertheless, there was still a possibility that some of our subjects may have asthma. As considering this, we additionally analyzed each data from only male or from subjects with prescription including LAMA, which is specific for COPD (Additional file 1: Table S1 and Table S2). Both analyses showed the same trend as Table 5. Since we used health insurance claim data, relatively important clinical data such as smoking history, lung function, and body mass index cannot be quantified although we tried to exclude patients with asthma or those with obesity or other diseases increasing risk of GERD by using both diagnosis codes and prescription data. Relatively not severe exacerbation such as unscheduled clinic visits or medication change were not included. Also, our definition of exacerbation might exclude some patients receiving only antibiotics without systemic corticosteroid. However, despite of these limitations, the present study clearly exhibits various interactive relationships between COPD and GERD. Although it would be hard to conclude complex relationship between COPD and GERD considering own severity and specific medications for both diseases and their interactions, we need to pay more attention to unraveling the heterogeneous causes of exacerbations.

## Conclusions

GERD is one of the most common comorbidities and a factor associated with exacerbation in patients with COPD. COPD-related medications except inhaled anticholinergics are associated with increased risk of GERD.

## Abbreviations

COPD: Chronic obstructive pulmonary disease; GERD: Gastroesophageal reflux disease; HIRA: Korean Health Insurance Review and Assessment Service; ICU: Intensive care unit; ER: Emergency room; ICS: Inhaled corticosteroid; LABAs: Long-acting beta-2 agonists; LAMAs: Long-acting muscarinic antagonists; SAMAs: Short-acting muscarinic antagonists; SABAs: Short-acting beta-2 agonists; OCS: Oral corticosteroid; LTRAs: Leukotriene receptor antagonists; OR: Odds ratio; CI: Confidence interval; PPIs: Proton pump inhibitors.

## Competing interests

YMO has been an investigator in university-sponsored studies (Asan Institute for Life Science, University of Ulsan College of Medicine) and an industry-sponsored study (MSD Korea and AstraZeneca Korea) and has participated as a speaker at scientific meetings organized and financed by various pharmaceutical companies (Handok, GlaxoSmithKline, AstraZeneca Korea, MSD Korea, and Boehringer Ingelheim) and a magazine company (Korea Doctors’ Weekly). SDL serves as a consultant to GlaxoSmithKline and has participated as a speaker at scientific meetings organized and financed by various pharmaceutical companies (GlaxoSmithKline, AstraZeneca Korea, and Boehringer Ingelheim). The remaining authors have no conflicts of interest to disclose.

## Authors’ contributions

JK participated in data analysis and drafted the manuscript. JHL conceived the study, participated in the study design and drafted the manuscript. YK and KK participated in the study design and performed data analysis. YMO, KHY, CKR, HKY, YSK, YBP, SWL, and SDL participated in the study design and were involved in revising the manuscript. All authors read and approved the final manuscript.

## Pre-publication history

The pre-publication history for this paper can be accessed here:

http://www.biomedcentral.com/1471-2466/13/51/prepub

## Supplementary Material

Additional file 1: Table S1 Association of GERD with exacerbation in male patients with COPD. **Table S2** Association of GERD with exacerbation in patients with COPD receiving LAMAs. Click here for file
