# Supplementary material for: Association between chronic obstructive pulmonary disease and gastroesophageal reflux disease: a national cross-sectional cohort study
Source: BMC Pulm Med. 2013 Aug 9;13:51. doi: 10.1186/1471-2466-13-51 (PMC3750392; doi:10.1186/1471-2466-13-51)
Supplement: Additional file 1: Table S1 — Association of GERD with exacerbation in male patients with COPD. Table S2 Association of GERD with exacerbation in patients with COPD receiving LAMAs. [file 1471-2466-13-51-S1.doc]

**Additional file**

**Table S1** Association of GERD with exacerbation in male patients with COPD

|  | | Hospitalization | | | ICU | | | ER visit (0-1 *vs*. ≥2) | | |
| --- | --- | --- | --- | --- | --- | --- | --- | --- | --- | --- |
|  |  | OR | 95% CI | *p* | OR | 95% CI | *p* | OR | 95% CI | *p* |
| **GORD** | No | 1 (ref) |  |  | 1 (ref) |  |  | 1 (ref) |  |  |
|  | Yes | 1.51 | (1.46, 1.56) | <0.001 | 0.96 | (0.77, 1.21) | 0.732 | 1.60 | (1.51, 1.70) | <0.001 |
| **GERD^*^** | No | 1 (ref) |  |  | 1 (ref) |  |  | 1 (ref) |  |  |
|  | Yes | 1.52 | (1.47, 1.57) | <0.001 | 0.95 | (0.76, 1.20) | 0.687 | 1.57 | (1.48, 1.66) | <0.001 |

ICU = intensive care unit; ER = emergency room; OR = odds ratio.

*Adjusted for sex, age, type of health insurance, and COPD severity.

**Table S2** Association of GERD with exacerbation in patients with COPD receiving LAMAs

|  | | Hospitalization | | | ICU | | | ER visit (0-1 *vs*. ≥2) | | |
| --- | --- | --- | --- | --- | --- | --- | --- | --- | --- | --- |
|  |  | OR | 95% CI | *p* | OR | 95% CI | *p* | OR | 95% CI | *p* |
| **GORD** | No | 1 (ref) |  |  | 1 (ref) |  |  | 1 (ref) |  |  |
|  | Yes | 1.57 | (1.50, 1.64) | <0.001 | 1.14 | (0.85, 1.52) | 0.393 | 1.53 | (1.43, 1.64) | <0.001 |
| **GERD^*^** | No | 1 (ref) |  |  | 1 (ref) |  |  | 1 (ref) |  |  |
|  | Yes | 1.56 | (1.49, 1.63) | <0.001 | 1.10 | (0.82, 1.48) | 0.516 | 1.50 | (1.40, 1.61) | <0.001 |

ICU = intensive care unit; ER = emergency room; OR = odds ratio.

*Adjusted for sex, age, type of health insurance, and COPD severity.
